# Use of Intensive Care Unit in Women with Severe Maternal Morbidity and Maternal Death: Results from a National Multicenter Study

**DOI:** 10.1055/s-0040-1708095

**Published:** 2020-03

**Authors:** Fabiano M. Soares, José Paulo Guida, Rodolfo Carvalho Pacagnella, João Paulo Souza, Mary Ângela Parpinelli, Samira Maerrawi Haddad, José Guilherme Cecatti

**Affiliations:** 1 Department of Obstetrics and Gynecology, Universidade Estadual de Campinas, Campinas, SP, Brazil; 2 UNDP/UNFPA/UNICEF/WHO/World Bank Special Programme of Research, Development and Research Training in Human Reproduction (HRP), Department of Reproductive Health and Research, WHO, Geneva, Switzerland

**Keywords:** intensive care unit, severe maternal morbidity, maternal near-miss, complications of pregnancy, Unidade De Terapia Intensiva, morbidade materna grave, *near-miss* materno, complicações da gravidez

## Abstract

**Objective** To assess the use of the intensive care unit (ICU) and its effect on maternal mortality (MM) among women with severe maternal morbidity (SMM).

**Materials and Methods** A secondary analysis of a cross-sectional study on surveillance of SMM in 27 Brazilian obstetric referral centers. The analysis focused on the association between ICU use and maternal death according to individual characteristics and disease severity. Two multivariate regressions considering use of the ICU, age, ethnicity, adequacy of care and the human development index were performed to identify the factors associated to maternal death and maternal near-miss.

**Results** Out of 82,388 deliveries during the period, there were 9,555 (11.6%) women with SMM, and the MM ratio was of 170.4/100 thousand live births. In total, 8,135 (85.1%) patients were managed in facilities in which ICUs were available; however, only 2,059 (25.3%) had been admitted to the ICU. On the multivariate analysis, when the severity of the maternal disease was measured by the maternal severity score (MMS), the strength of the association between the use of the ICU and maternal death was greatly reduced, along with inadequate care and non-availability of the ICU at the facility. On the assessment of only the more critical cases (SMO, severe maternal outcome), the same pattern of association between ICU and MM was observed. In the models used, only inadequate care and MSS were significantly associated with MM.

**Conclusion** The current study indicates that the main variables associated with maternal death are the severity and adequacy of the case management, which is more frequent in ICU admissions. The use of the ICU without the stratification of the patients by severity may not produce the expected benefits for part of the women.

## Resumo

**Objetivo** Avaliar o efeito da utilização de unidades de terapia intensiva (UTIs) na mortalidade materna (MM) entre mulheres com morbidade materna grave (MMG).

**Materiais e Métodos** Foi realizada uma análise secundária de um estudo transversal de vigilância de morbidade materna grave em 27 centros de referência obstétrica no Brasil. O foco desta análise foi a associação entre a utilização de UTI e morte materna segundo características individuais e condições de gravidade. Análises múltiplas considerando as variáveis uso de UTI, idade, etnia, adequação do cuidado e índice de desenvolvimento humano foram realizadas para identificar os fatores associados à morte materna e *near-miss* materno.

**Resultados** Dos 82.388 partos ocorridos durante o período de estudo, 9.555 (11,6%) mulheres apresentaram MMG, e a razão de MM foi de 170,4/100 mil nascidos vivos. Neste grupo, 8.135 (85,1%) pacientes foram atendidas em instituições com disponibilidade de leitos de UTI, mas apenas 2.059 (25,3%) foram de fato admitidas em leitos de UTI. Na análise de regressão multivariada, quando se considerou a gravidade do caso pelo *maternal severity score* (pontuação de severidade materna, MMS, na sigla em inglês), houve uma grande redução da força de associação entre utilização de UTI e morte materna, além da inadequação do cuidado e não disponibilidade de UTI na instituição. Na avaliação considerando apenas os casos de maior gravidade (desfecho materno grave, DMG), observou-se o mesmo padrão de associação entre UTI e MM. Nos modelos utilizados, apenas a inadequação do cuidado e o MSS apresentam associação significativa com a MM.

**Conclusão** O presente estudo aponta que as principais variáveis associadas à morte materna são a gravidade e a adequação do manejo do caso, mais frequentes nas internações em UTI. A utilização dos leitos de UTI sem a estratificação da gravidade da paciente pode não trazer benefícios esperados para uma parte das mulheres.

## Introduction

In the past ten years, the maternal mortality (MM) ratio in Brazil has remained stable. Approximately 70 women die per 100 thousand live births in the country,^1^ which places Brazil in the third stage of obstetric transition. In this stage, despite the still high MM ratios, a larger number of women are able to receive medical care during pregnancy, and the outcomes are strongly determined by the quality and accessibility of healthcare services.^2^

It is currently understood that maternal death (MD) is an event preceded by different potentially life-threatening conditions (PLTCs) that continue to worsen until some degree of dysfunction or organ failure occurs,^3^ if the treatment is inadequately instituted, both in terms of resources and time.^4^ ^5^ The woman who is affected by these conditions and survives, either due to adequate treatment or luck, is classified as a case of maternal near-miss (MNM). The World Health Organization (WHO) has defined MNM using clinical and laboratory criteria, or those associated with care.^3^

In general, PLTCs require care in more complex secondary or tertiary hospital units, while dysfunction and organ failure in cases of MNM obtain the most benefit from the ICU treatment. Regarding the total number of ICU admissions, the number of obstetric ICU admissions is small. However, certain particularities occur in the care of a woman during her pregnancy-puerperal cycle. Physiological and anatomical changes occur in the mother, along with changes related to the fetus. Early termination of pregnancy may also be required.^6^

It is intuitive to think that severe acute conditions, which characterize severe maternal morbidity (SMM), represented by PLTC and MNM, are better managed in the ICU. Nonetheless, it is known that the Brazilian public health system is not equipped with ICU beds and specialized professionals to deal with all the cases that occur in the country. Furthermore, factors associated with the successful or unsuccessful treatment of obstetric ICU cases and prioritization of conditions to provide differentiated care are largely unknown. There is no doubt that data obtained during ICU admission are precious tools for clinical research and also to understand the progression and behavior of diverse severe clinical conditions.^7^ ^8^

Therefore, the aim of the present secondary analysis is to assess the use of the ICU in women with severe maternal morbidity and its association with maternal mortality in Brazil.

## Materials and Methods

The present is a secondary analysis of a multicenter cross-sectional study conducted in various centers in Brazil. The study protocol was previously published,^9^ along with its main results.^10^ Briefly, during the study period, all women from 27 participating centers admitted with criteria for PLTCs, for MNM and MD were included in the study. The medical charts of those women were reviewed, and their data was inserted in a database developed in the OpenClinica (OpenClinica, LLC, Waltham, MA, US) electronic platform*.* Demographic, obstetric and clinical information were obtained from all women included, as well as data on their respective newborn infants. The information was stored in a safe system and the coordinating center analyzed data consistency and quality.

In the secondary analysis, all women with PLTC, MNM and MD were included. We considered the WHO criteria for maternal morbidity: SMM includes women with PLTC or MNM (SMM = PLTC + MNM) and severe maternal outcome (SMO) includes women with MNM and MD (SMO = MNM + MD), reflecting degrees of severity. Women were compared in terms of ICU availability and admission, and were divided into three groups: lack of ICU, availability of ICU without ICU admission, and ICU availability and admission.

The source of funds for hospital admission was evaluated (whether public or private), as well as the adequacy of the maternal treatment provided by the facility. All centers provided tertiary care. Furthermore, women were categorized according to age, skin color, marital status, schooling, and number of pregnancies. The maternal severity score (MSS) was also used, and it stratifies patient severity by adding scores related to severity.^11^ The human development index (HDI) of the city where the facility was located was also considered. This index considers life expectancy at birth, education and gross domestic product per capita, and its results vary from 0 (less developed) to 1 (more developed). The HDI enables the comparison of different geographical regions regarding social development.

The adequacy of care was assessed by the ratio between the expected number of deaths and the observed number of deaths in each health facility. The provision of care was considered adequate when the ratio was between 0.4 and 1.24, and inadequate when the ratio was higher than 1.25.^12^ For this variable, each woman received a classification of adequacy attributed to the respective healthcare facility where she was treated.

### Statistical Analysis

The existence and distribution of MNM criteria were categorized according to single or associated causes, and the complications experienced by women were classified as hypertensive, hemorrhagic, infectious and clinico-surgical.

Two multivariate analyses using Poisson regression were performed to estimate the association between MM and MNM with individual characteristics of patients and healthcare facilities, and ICU availability and admission, using the prevalence ratios (PRs) and their respective 95% confidence intervals (95%CIs) as measures of effect. Variables with a very high proportion of missing values were not included in the models. The analyses were carried out initially taking into account all cases of SMM as a reference and then considering only cases of MNM. The inclusion of distinct predictors was shown by stages in the model. In the first model, we included the use of ICU; in the second model, age and ethnicity were added; in the third model, adequacy of care and non-availability of the ICU were then included; in the fourth model, the MSS was added; and finally, in fifth model, the HDI was also added. The variables were defined after consulting the research group, which was composed by obstetricians, since there were no previous similar studies in literature. The statistical analyses were performed considering the cluster design effect, and the Statistical Package for the Social Sciences (SPSS, IBM Corp., Armonk, NY) software was used.

### Ethical Approval

The research protocol for the present study was approved by the Institutional Review Board (IRB) of the coordinating institution (document CEP 027/2009), as well as the local IRBs of each participating center. The need for individual informed consent was waived, since the data were only collected from clinical records after hospital discharge or death of each participating woman.

## Results

Out of the 82,388 births included in the study, 11.6% (9,555) of the women had some negative maternal outcome, SMM or MD. Furthermore, 1,420 (14.9%) women were managed in healthcare facilities lacking ICUs, 6,076 (63.6%) women were cared for in facilities equipped with ICUs, although they were not admitted to those units, and 2,059 (21.5%) patients were treated in the ICU. Intensive care units were available in 77.8% of the participating facilities. Furthermore, the frequency of ICU use was of 2.5%, considering all deliveries included in the study. Nevertheless, when only women with SMM were considered, the rate of ICU use was of 21.5%. Evaluating only women with SMOs, we observed that 64.5% of them had been admitted to the ICU. Nonetheless, the frequency of SMOs identified among women using the ICU was of 28.5%. In contrast, the proportion of MDs without ICU admission was of 17.1%. In addition, the total number of MDs inside the ICU was 116, representing 5.6% of the 2,059 women admitted in to ICU (Table 1).

**Table 1:** Indicators of intensive care units used for women with pregnancy-related conditions or those that occur during pregnancy

Intensive care unit (ICU) use	n(%)
Total of live births in the original study	82,388
Total number of women with severe maternal morbidity	9,555
Non-availability of ICU in the facility	1,420 (14.9)
Availability of ICU, but no admission	6,076 (63.6)
Availability of ICU, and admission	2,059 (21.5)
Proportion of ICU admissions that resulted in maternal deaths	116/2,059 (5.6)
Facilities in the study with ICU	77.8%
ICU admission rate per live birth in the original study	2.5%
ICU admission rate among cases of severe maternal morbidity	21.5%
ICU admission rate among women with severe maternal outcome	64.5%
Severe maternal outcome rate among women admitted to the ICU	28.5%
Proportion of maternal deaths assisted without ICU admission	17.1% (24)

Table 2 compares women according to ICU availability and admission. Most women were aged between 20 and 29 years, white and married. Almost half of the women included were in their first pregnancy. The majority (79.4%) of the women admitted to the ICU underwent treatment in facilities classified as having adequate maternal care, while only 42.5% of women treated in facilities without ICU were classified as receiving adequate care. Table 2 also shows that 100% of the hospitals lacking ICUs were public. None of these characteristics differed significantly among the three groups in terms of ICU availability and accessibility.

**Table 2:** Characteristics of women with complications by intensive care unit (ICU) status

Characteristics	No ICU availability n(%)	ICU availability, no admission n(%)	ICU availability, admission n(%)	Total n(%)
**N**	1,420 (14.9)	6,076 (63.6)	2,059 (21.5)	9,555
**Maternal deaths**	12 (0.8)	12 (0.2)	116 (5.6)	140
**Age**				
10-19	244 (17.2)	1,064 (17.5)	405 (19.7)	1713 (17.9)
20-29	688 (48.5)	2,925 (48.1)	943 (45.8)	4556 (47.7)
30-39	421 (29.6)	1,802 (29.7)	594 (28.8)	2817 (29.5)
40-49	67 (4.7)	285 (4.7)	117 (5.7)	469 (4.9)
*p*-value				0.557
**Ethnicity** ^a^				
White	663 (65.3)	2,413 (54.9)	1032 (59.8)	4108 (57.5)
Non-white	352 (34.7)	1,985 (45.1)	694 (40.2)	3031 (42.5)
*p*-value				0.662
**Marital status** ^b^				
With partner	435 (46.9)	2,676 (49.5)	1162 (68.0)	4273 (53.2)
Without partner	492 (53.1)	2,727 (50.5)	547 (32.0)	3766 (46.8)
p-value				0.083
**Schooling** ^c^				
Primary school	353 (48.8)	2,125 (45.5)	738 (48.2)	3216 (46.5)
High school	337 (46.5)	2,274 (48.7)	701 (45.8)	3312 (47.8)
University	34 (4.7)	270 (5.8)	91(5.9)	395 (5.7)
*p*-value				0.784
Number of pregnancies* ^d^				
One pregnancy	578 (41.7)	2,524 (41.6)	873 (42.8)	3975 (41.9)
2-3 pregnancies	517 (37.3)	2,367 (39.0)	755 (37.0)	3639 (38.3)
> 3 pregnancies	291 (21.0)	1,174 (19.4)	414 (20.3)	1879 (19.8)
p-value				0.721
**Type of hospital**				
Public	1420 (100)	5,500 (90.5)	1361 (66.1)	8281 (86.7)
Not public	0 (0.0)	576 (9.5)	698 (33.9)	1274 (13.3)
*p*-value				0.213
**Adequacy of care**				
Adequate	603 (42.5)	3,861 (63.5)	1634 (79.4)	6098 (63.8)
Inadequate	817 (57.5)	2,215 (36.5)	425 (20.6)	3457 (36.2)
*p*-value				0.413

Note: *Including the current pregnancy; missing: ^a^2,416; ^b^1,516; ^c^2,632; 4^d^62.

Most women evaluated did not meet the MNM criteria, although almost 30% of those admitted to the ICU had at least 1 near-miss criterion. Table 3 also shows that almost 10% of the women treated in hospitals lacking ICUs had near-miss criteria, while less than 4% of the women treated in hospitals with ICUs who were not admitted to these units had near miss criteria. These data suggest that patients with near-miss criteria treated in hospitals with available ICUs are more readily transferred to these units. Women with less severe conditions remain under the usual medical care.

**Table 3:** Distribution of criteria for the definition of maternal near-miss (MNM) according to the status of intensive care unit (ICU) use

Criteria for MNM	No ICU availability n(%)	ICU availability, no admission n(%)	ICU availability, admission n(%)	Total n(%)
None Clinical Laboratory Management Clinical + Laboratory Clinical + Management Laboratory + Management Clinical + Laboratory + Management *p*-value	1,300 (91.5) 36 (2.5) 12 (0.8) 32 (2.3) 05 (0.4) 23 (1.6) 02 (0.1) 10 (0.7)	5,873 (96.7) 30 (0.5) 78 (1.3) 49 (0.8) 06 (0.1) 15 (0.2) 11 (0.2) 14 (0.2)	1,472 (71.5) 47 (2.3) 82 (4.0) 94 (4.6) 27 (1.3) 85 (4.1) 29 (1.4) 223 (10.8)	8,645 (90.5) 113 (1.2) 172 (1.8) 175 (1.8) 38 (0.4) 123 (1.3) 42 (0.4) 247 (2.6) < 0.001

Table 4 shows that hemorrhagic and hypertensive complications were the ones most frequently found among the women evaluated. Nonetheless, there was no significant difference in these conditions regarding admission or not to the ICU. Infections and clinico-surgical conditions were significantly more common among cases that had access to the ICU. Patients admitted to the ICU had a higher rate of multiple complications (8.8%) than those who were not admitted to the ICU (4.7%) or those without an ICU available (5.6%).

**Table 4:** Distribution of women experiencing complications according to availability of and admission to the intensive care unit (ICU)

Complications		No ICU n (%)	ICU availability, no admission n (%)	ICU availability, admission n (%)	Total n (%)	*p*-value
Hemorrhage	No	1,065 (75.0)	4,502 (74.1)	1,710 (83.1)	7,277 (76.2)	0.323
	Yes	355 (25.0)	1,574 (25.9)	349 (16.9)	2,278 (23.8)	
Hypertensive disorders	No	371 (26.1)	1,858 (30.6)	620 (30.1)	2,849 (29.8)	0.836
	Yes	1,049 (73.9)	4,218 (69.4)	1,439 (69.9)	6,706 (70.2)	
Infections	No	1,413 (99.5)	6,039 (99.4)	2,003 (97.3)	9,455 (99.0)	< 0.001
	Yes	07 (0.5)	37 (0.6)	56 (2.7)	100 (1.0)	
Clinical and surgical complications	No	1,331 (93.7)	5,542 (91.2)	1,659 (80.6)	8,532 (89.3)	< 0.002
	Yes	89 (6.3)	534 (8.8)	400 (19.4)	1,023 (10.7)	
Number of causes	1	1,340 (94.4)	5,789 (95.3)	1,878 (91.2)	9,007 (94.3)	< 0.002
	2 or +	80 (5.6)	287 (4.7)	181 (8.8)	548 (5.7)	

Table 5 shows that access to the ICU alone had a PR of 17.6 in association with MD. However, as the variables previously described are added to the multiple analysis model, this PR decreased to 7.19, and inadequate care (PR = 3.38), non-availability of ICU (PR = 4.10) and MSS (PR = 1.44) also became independently associated with maternal death. Admission to the ICU was independently associated with a higher rate of MDs in diverse models that tested different variables. Regression models were applied, using the population of SMM cases as a reference (Table 5) or only cases of MNM (Table 6). In addition, the following predictors were included in sequence: admission to the ICU, maternal age, ethnicity, classification of adequacy of care in the facility, non-availability of the ICU, MSS and HDI of the state where the health unit was located. In contrast, the same effect was demonstrated among cases of MNM (Table 6). When the only variable assessed in the model of association with MD was the ICU admission, the PR found was of 2.66, while with the sequential addition of other variables, ICU admission was no longer associated with MD. Furthermore, inadequate care (PR = 2.57) and MSS (PR = 1.30) remained independently associated with the outcome of MD.

**Table 5:** Variables independently associated with maternal death among all cases of severe maternal morbidity using different models for multiple analysis by Poisson* regression

Model/Variable	Prevalence ratio	95% confidence interval	*p*-value
Model 0 [n = 9,555]			
- Used the intensive care unit (ICU)	17.60	8.51–36.39	< 0.001
Model 1 [n = 7,139]			
- Used the ICU	17.30	7.58–39.51	< 0.001
- Age (years)	0.99	0.95–1.03	0.662
- Color/ethnicity (white)	1.62	0.97–2.70	0.064
Model 2 [n = 7,139]			
- Used the ICU	43.31	17.74–105.74	< 0.001
- Age (years)	1.00	0.97–1.03	0.969
- Color/ethnicity (white)	1.47	0.90–2.38	0.117
- Adequacy of care (inadequate)	4.60	2.33–9.07	< 0.001
- Non-availability of the ICU	4.62	1.84–11.58	0.002
Model 3 [n = 7,139]			
- Used the ICU	8.04	3.57–18.10	< 0.001
- Age (years)	0.99	0.96–1.01	0.287
- Color/ethnicity (white)	1.53	1.02–2.29	0.042
- Adequacy of care (inadequate)	3.53	2.06–6.04	< 0.001
- Non-availability of the ICU	3.91	1.72–8.89	0.002
- MSS	1.43	1.39–1.47	< 0.001
Model 4 [n = 7,139]			
- Used ICU	7.19	3.05–16.95	< 0.001
- Age (years)	0.99	0.96–1.02	0.527
- Color/ethnicity (white)	1.36	0.93–1.99	0.104
- Adequacy of care (Inadequate)	3.38	2.00–5.71	< 0.001
- Non-availability of ICU	4.10	1.79–9.41	0.002
- Severe maternal morbidity	1.44	1.39–1.48	< 0.001
- Human development index (> 0.772)	1.41	0.70–2.83	0.320

Note: * Analysis considering cluster design effect (center).

**Table 6:** Variables independently associated with maternal death among cases of maternal near-miss using different models for multiple analysis by Poisson* regression

Model/Variable	Prevalence ratio	95% confidence interval	*p*-value
Model 0 [n = 910]			
- Used the intensive care unit (ICU)	2.66	1.73–4.09	< 0.001
Model 1 [n = 724]			
- Used the ICU	2.80	1.60–4.92	< 0.002
- Age (years)	0.98	0.95–1.01	0.123
- Color/ethnicity (white)	1.04	0.70–1.53	0.854
Model 2 [n = 724]			
- Used the ICU	4.77	2.39–9.52	< 0.001
- Age (years)	0.98	0.96–1.01	0.157
- Color/ethnicity (white)	1.05	0.72–1.53	0.781
- Adequacy of care (inadequate)	2.46	1.49–4.04	< 0.002
- Non-availability of the ICU	2.08	0.94–4.60	0.070
Model 3 [n = 724]			
- Used the ICU	1.70	0.84–3.41	0.131
- Age (years)	0.99	0.96–1.01	0.165
- Color/ethnicity (white)	1.27	0.92–1.75	0.137
- Adequacy of care (inadequate)	2.58	1.81–3.67	< 0.001
- Non-availability of the ICU	1.60	0.75–3.43	0.212
- Severe maternal morbidity	1.29	1.26–1.33	< 0.001
Model 4 [n = 724]			
- Used the ICU	1.63	0.78–3.37	0.183
- Age (years)	0.99	0.96–1.01	0.273
- Color/ethnicity (white)	1.20	0.90–1.60	0.199
- Adequacy of care (inadequate)	2.57	1.81–3.64	< 0.001
- Non-availability of the ICU	1.65	0.77–3.52	0.186
- Severe maternal morbidity	1.30	1.26–1.34	< 0.001
- Human development index (> 0.772)	1.19	0.77–1.85	0.412

Note: * Analysis considering cluster design (center).

## Discussion

The results of the present analysis show that not every referral obstetric service participating in the study had an available ICU, and that SMM affected around 10% of the women evaluated. Our results also demonstrated that ICU admission is independently associated with MD. However, the addition of other variables reduces and even abolishes this effect, particularly when evaluating this outcome exclusively among cases of MNM. First, it is important to clarify that the association between ICU admission and MD does not evidently imply causality. Admission to the ICU obviously did not cause MD. However, the health of the women deteriorated due to the sum of severe clinical conditions that also justified their need for ICU admission.

All centers included in the study were at least tertiary referral centers, and before their inclusion they were evaluated regarding their capacity to perform hysterectomy, mechanical ventilation, cardiorespiratory resuscitation in adults and newborn infants, and general anesthesia. They also were evaluated if they could provide resources for parenteral administration of antibiotics, oxytocin and magnesium sulphate, and we checked if all centers had a blood bank, obstetrical and neonatal ICUs, specialist care for high-risk pregnancies, availability of other medical or surgical specialties, ultrasonography, a laboratory, and anesthetists available round the clock. Not all centers had all of those resources; however, all centers included were considered referral centers for tertiary care in their geographic regions. The most important characteristic of the hospitals included in the present study was that the 27 centers are very representative of different regions of Brazil.

Our study demonstrated no significant differences regarding the sociodemographic characteristics of the patients admitted or not to an ICU. There were no ethnic disparities in access to healthcare services that had an ICU available, while the results in the literature^13^ have demonstrated that black women have less access to more qualified services. Furthermore, a systematic literature review^14^ showed that the lack of a partner was associated with worse outcomes during pregnancy. The present study showed that most women treated in a hospital lacking an ICU or who had not been admitted to the ICU were single. However, this difference was not significant.

Hypertensive and hemorrhagic disorders were the most prevalent conditions in the study population, reflecting the epidemiological profile of maternal health in Brazil. These disorders are most commonly associated with MD, which is in line with the period of obstetric transition that is taking place in the country.^15^ ^16^ Previous studies^17^ ^18^ have reported that hypertensive and hemorrhagic conditions are also the major causes of ICU admission, although our results revealed that clinical and surgical complications exceeded hemorrhagic conditions. A possible reason for this difference may be that the hospitals included in the present study are referral hospitals for severe cases in their areas. Referral hospitals manage a wider range of diseases during pregnancy that require advanced clinical support, while hemorrhagic complications may be rapidly managed by the obstetric team, avoiding the need to transfer patients to the ICU.

Multiple analysis of the association between ICU use and MM demonstrated that when only the variable ICU admission is considered, MD increases 17.60-fold (95%CI: 8.51–36.39) in the population of women with SMM, and 2.66-fold (95%CI: 1.73-4.09) in women with near-miss events. A probable explanation may be the transfer of more critically-ill patients to the ICU, where there is a concentration of women with more severe diseases and a higher risk of MD. This reinforces the non-existence of a causal relationship, although it is an indicator that a more critical and complex patient was admitted to the ICU. When MD death is controlled for facility characteristics, including adequacy of care, a decreased magnitude of the association between ICU use and MD is observed, indicating that lack of ICU or even inadequate ICU use is associated with an increased risk of MD.

Furthermore, after also controlling for an indicator of adequacy of care at the facility, in model 3, we found that the association between the risk of MD and ICU admission decreased. The decrease was around 2.5-fold in women with SMO, and 3.5-fold in women with SMM. This demonstrates that the main variables associated with MD are probably disease severity and patient management. The importance of stratifying the severity of the disease indicates that ICU use should be more effectively organized. The use of ICU without any clear criteria may not reduce the chance of MD as expected.

The tools for the systemization of risk stratification and optimal identification of women who would benefit the most from the use of ICU have been previously studied,^19^ along with specific instruments to classify maternal severity, such as the MSS.^11^ In this instrument, each marker is positively correlated with MM. As the number of markers increases, the probability of death increases, which may contribute to a better evaluation of the severity of obstetric populations, identifying women at increased risk for worse outcomes. In all of the models tested, the MSS was independently associated with MD, reinforcing the importance and usefulness of its implementation in the clinical practice.

## Conclusion

Admission to the ICU is associated with a higher rate of MD. The ICU receives more critically-ill patients as a result of both clinical conditions and inadequate care at the facility. Specific obstetric tools used to identify severity may help in the identification and early selection of cases that require attention in the ICU. This may decrease delays that cause the aggravation of clinical conditions and MDs.
